# Intra-Thyroid Thyroglossal Duct Cyst Incidentally Identified in an Adult With Primary Hyperparathyroidism: A Rare Case Report and Literature Review

**DOI:** 10.7759/cureus.20399

**Published:** 2021-12-13

**Authors:** Edward Chandraratnam, Juan Luo, Eva Wong

**Affiliations:** 1 Department of Anatomical Pathology, AustPath Laboratories, Northmead, AUS; 2 Department of Research and Development, AustPath Laboratories, Northmead, AUS; 3 Department of Surgery, Westmead Hospital, Westmead, AUS

**Keywords:** thyroglossal duct cyst, intra-thyroid, fine needle aspiration biopsy, cytology, histopathology, subtype

## Abstract

Thyroglossal duct cyst (TDC) commonly occurs in the neck just below the hyoid bone. Uncommon sites of TDC have been documented, and of these, an intra-thyroid location is very rare. We report such a rare intra-thyroid TDC (ITTDC) initially identified by ultrasound examination as an incidental thyroid imaging reporting and data system (TI-RADS) three lesion in the left thyroid lobe of a 59-year-old male patient with primary hyperparathyroidism due to a parathyroid adenoma. The preoperative ultrasound-guided fine-needle aspiration biopsy (US-FNAB) cytology of the thyroid lesion was interpreted as Bethesda III (atypia of undetermined significance or follicular lesion of undetermined significance). A left hemithyroidectomy and left superior parathyroidectomy were performed. The postoperative histology revealed the thyroid lesion to be an ITTDC. An incidental papillary thyroid microcarcinoma was also histologically revealed. The 2.5-year postoperative follow-up was uneventful. Based on literature searches, the clinical features, fine-needle aspiration biopsy (FNAB) cytology, histology, differential diagnosis, treatment, and follow-up of ITTDC were reviewed and discussed. A proposal to categorize ITTDC into two anatomical location subtypes is made. The liability of ITTDC to be misinterpreted on FNAB cytology due to rarity and lack of morphological specificity is emphasized.

## Introduction

Thyroglossal duct cyst (TDC) is among the most common cervical lesions encountered in children but not infrequent in adults [1]. TDC may theoretically occur anywhere along the traveling path of the developing thyroid gland and is typically found in the midline of the neck and around the level of the hyoid bone. An intra-thyroid location is one of those rare locations having been documented for TDC.

We report here such a very rare intra-thyroid TDC (ITTDC), which was incidentally detected as a cystic thyroid nodule during ultrasonographic workup for an adult patient with primary hyperparathyroidism. The cyst was preoperatively interpreted by ultrasound-guided fine-needle aspiration biopsy (US-FNAB) cytology as Bethesda III (atypia of undetermined significance or follicular lesion of undetermined significance) and subjected to hemithyroidectomy and histologically confirmed as an ITTDC.

Literature searches identified a very limited number of ITTDC cases documented in English medical literature [2-17]. Among them, those being reported as an incidental finding for other issues, like the present case, were extremely rare [3,13]. Based on the available data of reported cases, the clinical features, fine-needle aspiration biopsy (FNAB) cytology, histology, differential diagnosis, treatment, and follow-up of ITTDC were reviewed and discussed.

Consent for publication was obtained from the patient.

## Case presentation

History and auxiliary examination

A 59-year-old male patient presented with hypercalcemia (2.76 mmol/L) and elevated parathyroid hormone (PTH, 10.6 pmol/L) in 2016. Ultrasound examination revealed a 20 × 14 × 7 mm hypoechoic nodule, which was separate from and posterolateral to the left thyroid lobe. A parathyroid adenoma was suspected. Incidentally, a 7 × 4 mm hypoechoic nodule with at least one bright echogenic focus was identified in the left thyroid lobe. A sestamibi scan confirmed the parathyroid lesion. The serum thyroid-stimulating hormone (TSH) and free thyroxine (T4) levels were normal. Primary hyperparathyroidism and a left thyroid non-functioning nodule were clinically diagnosed. The interval blood tests during the following two years demonstrated persistently elevated levels of serum calcium (2.65 to 2.77 mmol/L) and parathyroid hormone (PTH, 11.8 to 13.9 pmol/L) and a normal level of TSH (1.19 mIU/L). In October 2018, a repeat ultrasound examination indicated an interval increase of the parathyroid lesion to a maximum diameter of 23 mm. The previously identified intra-thyroid lesion in the left lobe was measured 9 × 8 × 4 mm and categorized as thyroid imaging reporting and data system (TI-RADS) three.

US-FNAB cytology

**Figure 1:**
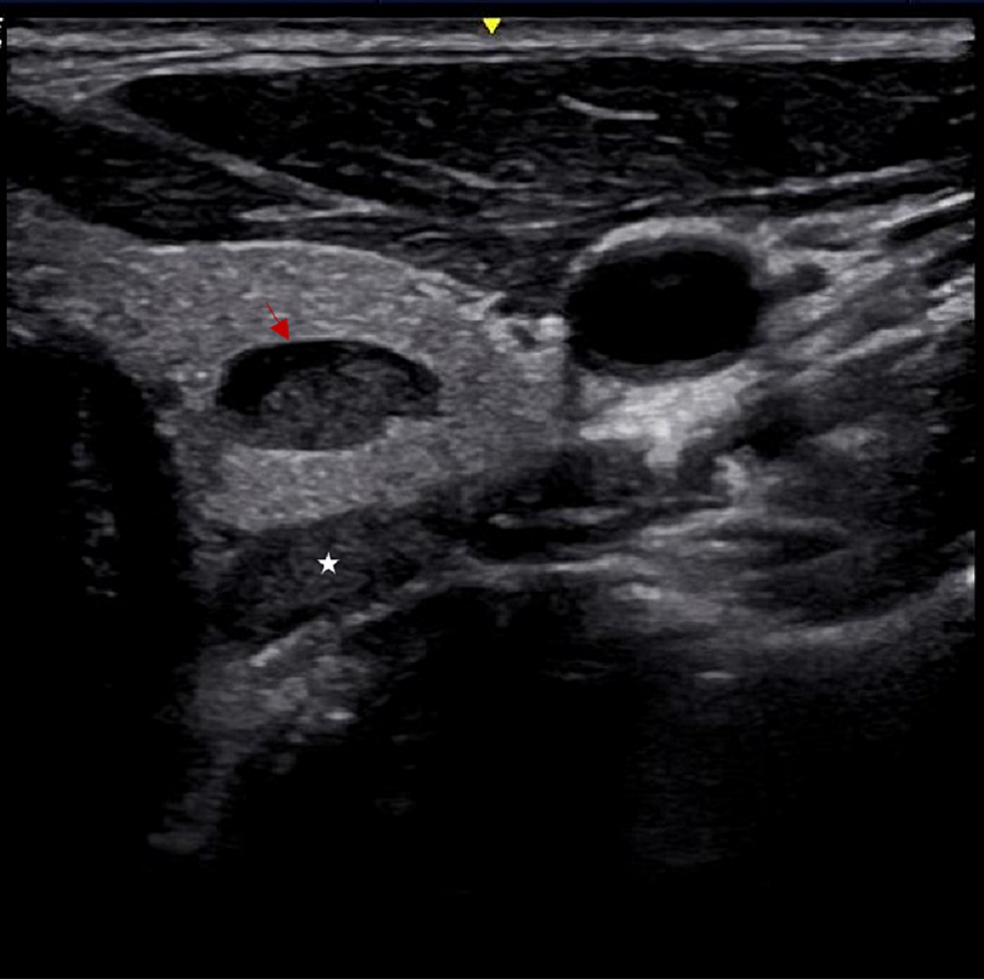
Ultrasound image of the left thyroid area during US-FNAB An ultrasound examination during US-FNAB revealed a hypoechoic cystic lesion (red arrow) in the left thyroid. The margins were smooth and well-defined. A few echogenic granules were observed in the capsule. An FNAB was then performed with two needle passes of a 21-gauge needle. A hypoechoic solid lesion (white star) corresponding to the site of the left parathyroid was also identified but not biopsied. US-FNAB - ultrasound-guided fine-needle aspiration biopsy

Preoperative US-FNAB of the predominantly cystic lesion in the left thyroid (Figure 1) produced a few drops of thick dark brown fluid. Following drainage, the lesion reduced in size by approximately 50%.

**Figure 2:**
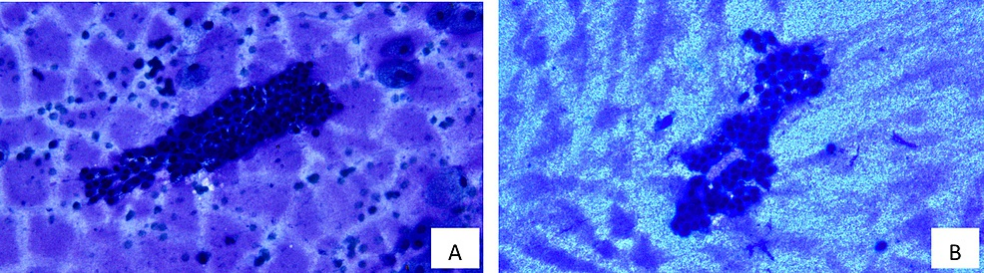
Cytology of US-FNAB of the left thyroid lesion 4A and 4B US-FNAB of the left thyroid lesion obtained three fragments of cohesive three-dimensional cell clusters in a background of thick granular proteinaceous precipitate (Diff-Quik staining at 400× original magnification). US-FNAB - ultrasound-guided fine-needle aspiration biopsy

The smears contained a thick granular proteinaceous precipitate, abundant old blood, and three epithelial fragments (Figure 2A, 2B). The epithelial cells were in cohesive three-dimensional clusters with scanty, optically dense, and pale cyanophilic cytoplasm. The nuclei were mildly enlarged with coarse granular chromatin. No nucleoli, significant pleomorphism, or mitotic figures were observed. Nuclear crowding and overlapping, irregularity of the nuclear membrane, and intranuclear cytoplasmic inclusions were not observed. No calcific material was identified. In view of the documented history of suspected parathyroid adenoma, a needle rinse of FNAB was submitted for hormone assays and returned with thyroglobulin of 13,900 ug/L, TgAb of < 1.0 klU/L, and intact PTH of < 4.0 ng/L. The FNAB cytology was interpreted as Bethesda III (atypia of undetermined significance or follicular lesion of undetermined significance).

Surgical operation

A left superior parathyroidectomy for the suspected parathyroid adenoma was performed. The Bethesda III lesion in the left thyroid lobe was managed by a left hemithyroidectomy. During operation, the surface of the left thyroid lobe looked intact. No duct-like structures or external adhesions were noticed.

Histopathology examination

On grass examination, a 15 × 11 × 7 mm in size, well-defined monocytic nodule was identified within the thyroid tissue (Figure 3.1). The surrounding thyroid tissue was within normal limits.

**Figure 3:**
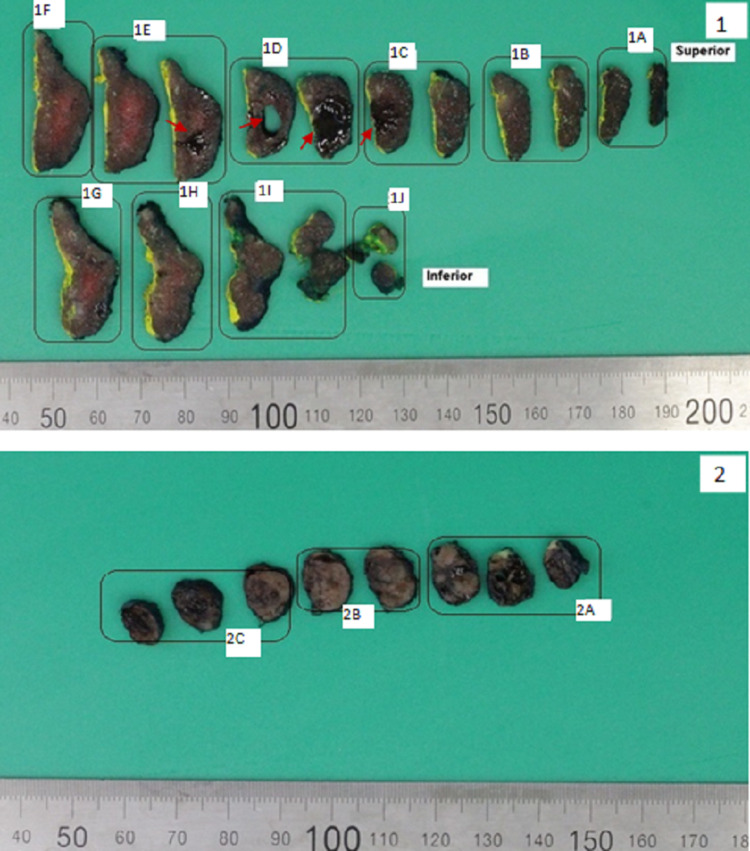
Gross examination of the left hemithyroidectomy and left superior parathyroid specimens (serially sliced) 3.1 A well-defined cyst filled with reddish-brown and thick fluid was identified (red arrows) in the mid-zone of the left hemithyroidectomy specimen. 3.2 The “left superior parathyroid” specimen presented as a solid oval nodule with dark tan and focally dark brown cut surface. No residual normal parathyroid tissue was macroscopically identifiable.

The left superior parathyroid was sent separately as an oval nodule with a smooth surface and measured 25 × 20 × 8 mm, and weighed 2.00 g (Figure 3.2). 

**Figure 4:**
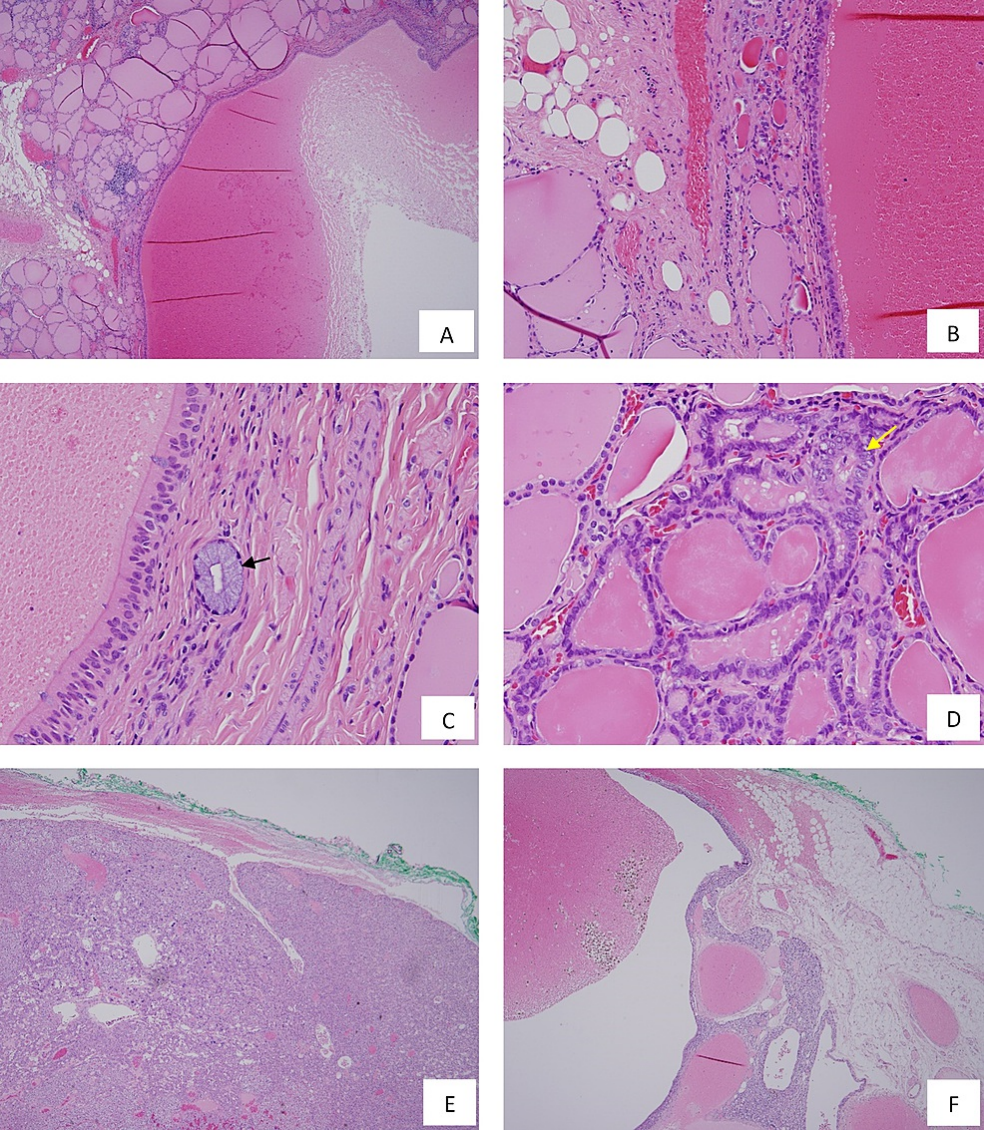
Microscopic examination of the left thyroid and left superior parathyroid lesions (H&E staining) 4A The intra-thyroid cyst filled with proteinaceous precipitate and surrounded by thyroid tissue (40× original magnification). 4B Higher magnification of the cyst with surrounding compressed thyroid parenchyma and adjacent adipose tissue (200× original magnification). 4C Bronchial type of respiratory lining epithelium of the cyst and the mucinous gland embedded in the cyst wall (black arrow, 400× original magnification). 4D The incidental PTMC with enlarged and crowded nuclei and nuclear grooves (yellow arrow, 400× original magnification). 4E Parathyroid adenoma showing predominant chief cells arranged in solid sheets with focal follicle formation (40× original magnification). 4F Cystic changes of the parathyroid adenoma (40× original magnification). H&E - hematoxylin and eosin, PTMC - papillary thyroid microcarcinoma

Microscopically, the intra-thyroid cyst (Figures 4A-4C) was filled with a thin proteinaceous precipitate. The lining epithelium was respiratory type (pseudostratified, cuboidal to columnar, and ciliated). The cyst wall was composed of a thin rim of hypocellular fibrocollagenous tissue merging with the surrounding compressed and atrophic thyroid parenchyma. A histology diagnosis of ITTDC was then made. One 0.1 mm oval gland (Figure 4C) lined by mucin-laden glandular cells with small, regular, and basally placed nuclei was also observed in the fibrocollagenous wall of the cyst. Lymphocytic thyroiditis of mild severity and early nodular hyperplasia were identified in the rest of the thyroid tissue.

During the preparation of the manuscript, to exclude any other pathological changes, the entire specimen was submitted for further histology examination, and an incidental papillary thyroid microcarcinoma (PTMC, 1.1 mm, Figure 4D) located away from the ITTDC was identified. The entire lesion of PTMC was confined to the thyroid (pT1a) and completely excised.

The left superior parathyroid nodule was histologically confirmed as a parathyroid adenoma which was composed of solid sheets of predominant chief cells with focal follicle formation and cystic changes (Figures 4E, 4F).

Follow-up

On the first postoperative day, the serum PTH level dropped sharply to the normal limit of 1.6 pmol/L, which demonstrated a successful surgical outcome. Since then (2.5 years postoperative), the patient has remained well with normal serum calcium and phosphate levels and without recurrence of the thyroid lesions.

## Discussion

During embryonic development, the thyroid anlage originates at the base of the tongue and moves down in the midline of the neck, forming an epithelial tract called the thyroglossal duct (TD). The thyroid gland forms within the distal portion of this duct. The duct then normally disappears. Rarely portions of the duct remain, which may accumulate fluid resulting in the formation of TDC [1, 3]. Autopsy studies of serially step-sectioned laryngeal specimens documented “islands of thyroid cells, ductal epithelium and cysts” in 7% (14/200 cases) of adults [18] and “complete thyroglossal tract” (4/58 cases) and “ectopic thyroid tissue” (20/58 cases) in 41.3% (24/58 cases) of children [19].

Intra-thyroidal location of TDC is rare. The exact incidence of ITTDC is unknown. In a large case series of TDC, Thompson et al. [1] observed 11/685 cases (1.6%) of TDC located within the thyroid gland. Further literature searches in PubMed and Google search identified 21 available cases of ITTDC reported in detail in English medical literature [2-17] (Table 1). The clinical features, FNAB cytology, histology, differential diagnosis, treatment, and follow-up of ITTDC were reviewed, summarized, and discussed based on these published cases.

**Table 1:** Documented ITTDC cases in the English medical literature Pub. year - year of publication, L - left, R - right, F - female, M - male, US - ultrasound examination, NA - not available, CW - consistent/compatible with, TDC - thyroglossal duct cyst, TD - thyroglossal duct, FS - frozen section, FFPE - formalin-fixed paraffin-embedded, CT - computed tomography, G+ - Gram positive, BG - background, ITTDC - intra-thyroid thyroglossal duct cyst

No.	Author	Pub. year	Country	Age (y)	Gender	Site	Clinical findings	Size (cm)	Biopsy & cytology	Surgery	Anatomical location subtype	Histology	Follow-up
1	Sonnino et al. [2]	1989	Canada	4	F	R	Thyroid nodule, cold on scan, fluid-filled mass on US	2	NA	R lobectomy + Sistrunk	II (within thyroid lobe with extension to the hyoid bone)	Multilocular cyst with features CW a TDC	4 yeas, no recurrence
2	Sonnino et al. [2]	1989	Canada	9	F	L	Hypofunctioning nodule on thyroid scan	3	NA	cyst excision	I (in the thyroid)	Lobulated cyst CW a TDC	3 years, no recurrence
3	North et al. [3]	1998	USA	58	M	R	L carotid bruit, incidentally identified a hypoechoic “solid” nodule in R thyroid on Duplex examination	1.1	US-FNAB: bloody; follicular & squamous cells, negative for malignancy	R lobectomy + isthmusectomy	I (in the thyroid, no evidence of TD)	Squamous mucosa-lined cyst	Period NA, no recurrence
4	North et al. [3]	1998	USA	78	M	Isthmus + R	Neck nodule becoming tender, discomfort with swallowing	2.5	FNAB: thick, beige colored, purulent appearing material; abundant normal appearing squamous cells	R lobectomy + isthmusectomy	I (within isthmus and extending into R thyroid lobe, no evidence of TD or pyramidal lobe)	Epidermal-lined cyst	Period NA, no recurrence
5	Hatada et al. [4]	2000	Japan	50	F	R	Lateral neck mass, discomfort on swallowing; cold on thyroid scan; low echoic mass on US	4.4	US-FNAB: thick, viscous, greyish fluid; normal appearing squamous cells, no follicular cells	R lobectomy	I (completely surrounded by thyroid tissue, no evidence of TD remnants)	Squamous epithelium-lined cyst	Period NA, no recurrence
6	Johnston et al. [5]	2003	USA	10	M	L	Congenital anterior neck mass, palpable cyst in L thyroid, cold on thyroid scan, cystic on US	3.5	“Needle aspiration”: tan & mucoid material; CW a TDC	L hemithyroidectomy + isthmusectomy	I (completely embedded within the thyroid gland, no TD was noted)	FS: benign development remnant; FFPE: alternating respiratory & squamous epithelia-lined cyst, paucity of lymphoid tissue in the subepithelial region	18 months, no recurrence
7	Roy et al. [6]	2003	India	50	F	R	Lateral neck swelling, clinically indistinguishable from solitary thyroid nodule; cystic on US	3	FNAB: a large number of nucleated squamous cells and anucleate squames	R hemithyroidectomy	I (intra-glandular, no evidence of TD extending from the thyroid)	Squamous epithelium-lined cyst with thyroid follicles in the cyst wall	4 years, no recurrence
8	Pérez-Martínez et al. [7]	2005	Spain	11	M	R	Visible neck mass, cold on thyroid scan, cystic on US	1.7	“Biopsied”: obtained mucus and cells	Cyst excision	I (the superior half of R thyroid was replaced by the cyst; no adjacent fistulous tract or tributary was found)	Non-keratinized squamous epithelium and mono-stratified mucinous epithelium-lined cyst, islets of thyroid tissue in the wall	8 months, no recurrence
9	Pueyo et al. [8]	2008	Spain	7	M	L	Neck nodule and upper respiratory tract infection; cold on thyroid scan, cystic on US	2.5	FNAB: mucoid material with squamous cells	Cyst excision + Sistrunk	II (with ascending tract connecting another 1.5 cm cyst below hyoid cartilage)	Both cysts and connecting tract lined by squamous epithelium with cylindrical and ciliated areas of respiratory type	NA
10	Álvarez Garcia et al. [9]	2015	Spain	2	M	R	Painless lateral neck mass, cold on scintigraphy, cystic on US	1.7	Not done	“The lesion was surgically resected”	I (in the thyroid)	Mucinous contenting cyst in the upper right lobe of the gland	NA
11	Álvarez Garcia et al. [9]	2015	Spain	10	M	R	Painless lateral neck mass; cold on scintigraphy, cystic on US	2	FNAB: squamous epithelium, absence of colloid material or follicular tissue	Cyst excision	I (in the R thyroid)	Non-keratinized squamous epithelium-lined cyst, proteinaceous material inside	NA
12	Huang et al. [10]	2015	China	45	F	L	Bilateral neck mass along the midline; cystic on US	4	Not done; instead, FS reported as a TDC	L hemithyroidectomy	I (separated nodule in L inferior pole of thyroid, no TD noted)	Pseudostratified ciliated columnar and squamous epithelia-lined cyst	NA
13	Saadi et al. [11]	2015	USA	48	M	Isthmus	Painless midline neck mass; cystic on US & CT	1.1	FNAB: benign epithelial cells & macrophages	Cyst excision + Sistrunk	II (connected with a TD traced superiorly to the hyoid bone)	Epithelia-lined cyst with a thin, fibrous extension to the hyoid bone containing thyroid follicles	1 week, no recurrence
14	Barber et al. [12]	2018	USA	36	M	L	Acute thyroiditis with a tender neck mass; L neck mass on CT, thyroid complex cyst on US	5	FNAB: thick purulent material; acute inflammation, lymphohistiocytic tangles, bland appearing follicular cells; G+ cocci	L lobectomy	I (completely surrounded by thyroid tissue, with no external tract present)	Predominantly respiratory (ciliated, pseudostratified columnar) epithelium-lined cyst with focal squamous metaplasia and chronic inflammatory reaction	NA
15	Handra-Luca et al. [13]	2018	France	36	F	NA	Hyperthyroidism, Graves’ disease	0.5	NA	Thyroidectomy for Graves’	I (incidentally identified under microscope)	Squamous or non-descript cells (with rare interspersed ciliated cells)-lined microcyst surrounded by hyperplastic adenoma-type follicular nodule; BG tissue: Graves’ with several dispersed ectopic tissue (skeletal muscle, thymic tissue, parathyroid tissue and adipose tissue)	NA
16	Handra-Luca et al. [13]	2018	France	NA	NA	NA	Hyperparathyroidism	0.4	NA	Thyroidectomy for “multinodular goiter during surgery for hyperparathyroidism”	I (incidentally identified under microscope)	Flat, mainly spindle-shaped epithelial cells-lined microcyst with rare ciliated cells and surrounded by fibrous tissue; L P4-neck parathyroid multifocal nodular hyperplasia; L P3-neck a thymus-parathyroid unit	NA
17	Lakshmi et al. [14]	2019	India	55	M	R	Anterior neck swelling; R thyroid cyst on US	2.7	FNAB: Scattered follicular epithelial cells and colloid in a hemorrhagic background, suggested to be a nodular colloid goiter, Bethesda II	Total thyroidectomy	I (intra-thyroid cyst, no tract identified)	Cyst lined by ciliated columnar, stratified squamous and flattered epithelium; fibrocollagenous tissue with thyroid follicles and scattered lymphoplasmacytic infiltrate in the cyst wall. BG tissue: nodular colloid goiter	NA
18	Prabha et al. [15]	2020	India	25	F	L	Neck lump, gradually progressing in size; large thyroid cyst on US	3.01	FNAB: dispersed benign squamous cells, few small sheets of follicular epithelial cells, numerous macrophages, colloid and neutrophils	L hemithyroidectomy	I (cystic lesion with surrounding thyroid tissue, no fistulous tract from the thyroid lobe)	Fibrocollagenous cyst wall lined by granulation tissue, hemosiderin laden macrophages and luminal anucleate squamous cells	9 months, no recurrence
19	Prabha et al. [15]	2020	India	41	M	L	Neck lump; large thyroid cyst on US	4.9	FNAB: paucicellular smears with dispersed mature benign squamous cells and few anucleate squames on a clean background	L hemithyroidectomy	I (cystic ballooned out nodule in the left lobe of thyroid)	Fibrocollagenous cyst lined partly by cuboidal epithelium with predominantly denuded lining; thyroid follicles, thin blood vessels and chronic inflammation in the cyst wall	6 months, no recurrence
20	Hakeem et al. [16]	2020	India	13	F	R + isthmus	Recurrent episodes of lower neck pain and swelling; cyst involving the R thyroid lobe and extended into the isthmus with intra-cystic calcified debris on US	4.3	FNAB: thick, viscous, dirty white colored fluid; lympho-histiocytic tangles and bland-appearing follicular cells	R lobectomy + isthmusectomy	I (completely surrounded by normal thyroid tissue, with no external tract present)	Cyst lined predominantly by pseudo-stratified ciliated columnar epithelium with focal squamous metaplasia and mild infiltrate of lymphocytes in the wall; secondary changes of chronic inflammation	NA, no recurrence
21	O’Neill et al. [17]	2021	USA	14	M	L	Neck swelling; a midline complex cystic structure near the L isthmus on US; a thick-walled, septated, complex fluid collection to the L of midline underneath the hyoid bone and extending inferiorly involving L lobe of thyroid near the junction of isthmus on CT	5	NA	L hemithyroidectomy + Sistrunk	II (cyst involving both L thyroid lobe and hyoid bone)	Cyst lined by pseudostratified ciliated columnar epithelium with focal squamous epithelium	6 months, no recurrence
22	Present case	NA	Australia	59	M	L	Hyperparathyroidism; incidentally identified a cystic lesion in L thyroid on US	1.5	US-FNAB: thick dark brown fluid; three epithelial fragments with no specific features in thick granular proteinaceous precipitate and abundant old blood	L hemithyroidectomy; L superior parathyroidectomy	I (completely within the left thyroid, no evidence of TD)	Cyst lined by pseudostratified, cuboidal to columnar and ciliated respiratory epithelium with one single small mucinous gland embedded in the hypocellular fibrocollagenous wall; L parathyroid gland: hypercellular parathyroid CW adenoma	2.5y, no recurrence

Clinical features of ITTDC

Although TDC has been reported predominantly in children, a wide range of age at diagnosis (0.8 to 87 years, mean age of 31.3 years with 62% of cases ≥20 years) was observed in Thompson et al.’s [1] case series. The published ITTDC cases (Table 1) were diagnosed at a mean age of 30.1 years (2 to 78 years, median age of 30.5 years) with 55% (11/20) ≥20 years old. More male patients (F:M=8:12) of ITTDC were documented, while no gender preponderance was shown in pooled age groups of TDC patients [1]. The present case of ITTDC is a male patient in his fifties.

As shown in Table 1, the greatest dimension of ITTDC ranged from 0.4 to 5 cm (mean of 2.8 cm, median of 2.7 cm). While most of these cysts were primarily presented as a neck/thyroid nodule or mass, the cysts in three cases were incidentally revealed during the imaging or histology workup for other issues (carotid bruit [3], hyperthyroidism [13], and hyperparathyroidism due to parathyroid multifocal nodular hyperplasia in a patient with multinodular goiter [13]). The small size (1.1, 0.5, and 0.4 cm, respectively), non-superficial intra-thyroid location, and concurrent multinodular goiter background may contribute to the asymptomatic existence of these ITTDCs. The present case was incidentally identified during the ultrasonographic workup for hyperparathyroidism caused by a parathyroid adenoma and initially measured less than 1 cm.

ITTDC can involve any lobe of the thyroid gland, with 42% (8/19), 42% (8/19), 5% (1/19), and 11% (2/19) being documented in the left, right, isthmus, and both isthmus and the right lobe, respectively (Table 1). ITTDCs in the pyramidal lobe were also noticed in Thompson et al.’s case series [1].

Interestingly, the specific anatomical relationship of ITTDC with the thyroid gland varies. We here propose to categorize ITTDC into two subtypes based on their relative anatomical location to the thyroid. Subtype I refers that the cyst being completely located within the thyroid gland without any form of extension outside of the thyroid capsule. Subtype II refers that the cyst is located at least partially within the thyroid gland with any form(s) (such as thyroid dysgenesis (TD) remnant, TD tract, and cyst) of external extension outside of the thyroid capsule. As such, 81% (17/21 cases) of the reported ITTDC can be categorized as anatomical location subtype I, with the four rest (19%) as subtype II (Table 1). The present ITTDC was completely located within the left thyroid lobe and can be categorized as subtype I.

FNAB cytology of ITTDC

Preoperative FNAB was documented for 13 ITTDC cases and eight of which were reported as squamous cells on cytology (Table 1). No definite diagnostic features were reported.

In the present case, dark brown fluid was drained during US-FNAB, which caused a marked reduction in the cyst size. The FNAB cytology was interpreted as abnormal (Bethesda III, atypia of undetermined significance or follicular lesion of undetermined significance) mainly due to the mild nuclear enlargement, three-dimensional cell crowding of the limited number of epithelial fragments, and the high level of needle rinse thyroglobulin. Even in a retrospect review of the FNAB cytology slides, no definite features indicating an ITTDC with bronchial type respiratory epithelial lining can be identified. Misinterpretation of the pseudostratified ciliated epithelium as atypical is well recognized in the cytology of the uterine cervix [20].

No literature was identified regarding the needle rinse thyroglobulin for ITTDC. The high level of thyroglobulin in the needle rinse of the present case may be due to either the contamination from the thyroid tissue on the needle pass or the production of ITTDC itself.

Histology of ITTDC

Histologically, the vast majority of general TDCs were reported to be lined by either respiratory (38%) or squamous (10%) epithelium or a combination of both (51%) [1]. However, squamous epithelium, followed by a combination of both types and respiratory epithelium only, was the most reported lining of ITTDC (Table 1). The present ITTDC was lined by bronchial type respiratory epithelium only and surrounded by compressed thyroid tissue like most of the reported ITTDC cases. Interestingly, one single mucinous gland was seen in the hypocellular fibrous cyst wall of the present case. Mucoserous salivary gland tissue was observed within the cyst wall in 15% (104/685) of TDCs [1].

The incidence of malignancy occurring in TDCs has been reported as 1% to 7.4% [1]. No such cases associated with ITTDC were identified in the English medical literature. In the present case, an incidental PTMC (1.1 mm) located away from the ITTDC was identified.

Differential diagnosis of ITTDC

Under physical and ultrasound examination, the intra-thyroid location of ITTDC mimics common thyroid lesions, such as nodular goiter and tumors with cystic changes. Although the cytology of ITTDC is non-diagnostic, FNAB can be used to rule out other lesions or identify malignancy. A supplementary needle rinse assay for PTH, like in the present case, can help exclude the rare possibility of intra-thyroid cystic parathyroid lesions, especially in the scenario of hyperparathyroidism. The histological appearance of ITTDC may overlap with another rare lesion described as an intra-thyroid lymphoepithelial cyst or branchial cleft-like cyst. Unlike ITTDC, intra-thyroid lymphoepithelial cysts are lined predominantly by squamous epithelium and consistently contain dense nodular or diffuse lymphoid infiltrate within the cyst wall [1].

Treatment and follow-up of ITTDC

The vast majority of the reported ITTDCs were located within the thyroid substance without evidence of TD remnants or external extension (anatomical location subtype I) and underwent cyst excision, lobectomy, or thyroidectomy only without Sistrunk procedure (Table 1). Four cases with external extension or connecting with a TD (anatomical location subtype II) underwent a supplementary Sistrunk procedure (Case 1, 9, 13, and 21). The limited follow-up data showed no recurrence of TDC one week to four years after surgery (Table 1). The present case (anatomical location subtype I) underwent hemithyroidectomy only, and no recurrence was observed during a 2.5-year follow-up.

## Conclusions

ITTDC is a very rare cystic thyroid lesion occurring in a wide range of ages and not infrequently found in adults. It can present as a neck/thyroid mass or an incidental finding on radiological or histological examination for unrelated thyroidal and/or non-thyroidal conditions. Aspiration of benign squamous cells, glandular cells, or a combination of both should alert the cytologist to the possibility of ITTDC. However, it appears that there are no specific cytological features to enable a definite preoperative FNAB diagnosis of ITTDC, and hence ITTDC will be a histological diagnosis.
